# Pilot application of an inflammation and physiological dysregulation index based on noninvasive salivary biomarkers

**DOI:** 10.1186/s13104-024-07056-4

**Published:** 2025-02-05

**Authors:** Andrey I. Egorov, William Xue, Jason Kobylanski, Miyu Fuzawa, Shannon M. Griffin, Timothy J. Wade, Michael Nye

**Affiliations:** 1 Office of Research and Development, U.S. Environmental Protection Agency, Chapel Hill, NC USA; 2 ORAU Contractor to the U.S. Environmental Protection Agency, Chapel Hill, NC USA; 3 Office of Research and Development, U.S. Environmental Protection Agency, Cincinnati, OH USA; 4 Office of Research and Development, U.S. Environmental Protection Agency, Denver, CO USA

**Keywords:** Physiological dysregulation, Inflammation, Allostatic load, Saliva, Biomarkers, Stress, Allergy, Neuroendocrine system, Immune system

## Abstract

**Background and objective:**

Noninvasive salivary biomarkers can be used to assess the cumulative subclinical health impacts of social and environmental stressors. We evaluated seven salivary biomarkers of neuroendocrine and immune functions and a group index of physiological dysregulation based on these biomarkers in a pilot survey in a socioeconomically disadvantaged population.

**Results:**

Participants recruited at subsidized public housing projects in Denver, Colorado, completed a sociodemographic, behavioral, and health questionnaire and used passive drool samplers to collect five saliva samples over the course of 1 day. Samples were tested for the neuroendocrine biomarkers alpha-amylase, cortisol, and dehydroepiandrosterone (DHEA) as well as immune system/inflammation biomarkers C-reactive protein (CRP), interleukin (IL)-1β, IL-6 and total immunoglobulin A (IgA). A composite Inflammation and Physiological Dysregulation Index (IPDI) was calculated as a count of potentially unhealthy values of dichotomized biomarkers. In 20 individuals who completed the survey (average age 46 years, 75% females), allergy to house dust was significantly associated with increased IPDI (adjusted for age) and significantly increased odds of potentially unhealthy values of total IgA, IL-1β, and CRP. Age, obesity, diabetes, allergy to pollen, recent loss of employment, and depression, anxiety, and stress scores from the standard DASS-21 questionnaire were positively but not significantly (0.05 < p < 0.2) associated with IPDI. This project demonstrated an application of a composite index based on noninvasive salivary biomarkers to assess subclinical health impacts of chronic social stressors.

**Supplementary Information:**

The online version contains supplementary material available at 10.1186/s13104-024-07056-4.

## Introduction

Noninvasive saliva sampling is better tolerated by survey participants, including children, than more invasive venous blood sampling approaches [1]. Saliva surveys by mail have been successfully conducted in the US [2–6] and other countries [7, 8]. Saliva samples can be used to measure certain biomarkers of neuroendocrine function and inflammation [9–12]. This pilot study assessed the feasibility of using self-collected saliva samples for analysis of selected biomarkers of neuroendocrine and immune functions reflecting the physiological impacts of chronic stress in economically disadvantaged communities. The specific aims were: (i) to evaluate the utility of the unsupervised saliva sampling approach and (ii) to explore associations of self-reported stress factors and health conditions with individual biomarkers and a composite Inflammation and Physiological Dysregulation Index (IPDI).

The IPDI index is related to the extensively described concept of Allostatic load (AL). AL is a composite measure of physiological dysregulation or “wear-and-tear” due to chronic stress [13–15]; it is strongly predictive of systemic morbidity and mortality [16, 17]. A typical AL group index represents a count of potentially unhealthy values of various biomarkers of immune, neuroendocrine, metabolic, and cardiovascular systems which are dichotomized at health-based or distribution-derived cutoffs, such as 25th or 75th percentile of the sample distribution, depending on which tail of the distribution is associated with an adverse health outcome [13]. Typical AL biomarkers, except salivary cortisol, are based on serum or urine samples or physical examinations. Chronic stress and environmental pollution can also cause chronic low-level systemic inflammation [18], which is a well-known risk factors for systemic diseases [19, 20]. A composite index of systemic inflammation has been used to characterize cumulative sub-clinical health impacts of various social and environmental factors along with AL [21].

The present study pilot-tested a saliva-based IPDI index based on seven well-established salivary biomarkers known to reflect the impacts of chronic stress. This IPDI index lacked some core stress-related AL biomarkers that cannot be measured in saliva, such as high-density and low-density lipoprotein, triglycerides, blood pressure, and many pro-inflammatory cytokines and other biomarkers of inflammation. Specifically, this study utilized three biomarkers of neuroendocrine function, α-amylase, cortisol, and dehydroepiandrosterone (DHEA), and four salivary biomarkers of immune function, C-reactive protein (CRP), interleukin (IL)-1β, IL-6, and total immunoglobulin A (IgA). Three of these biomarkers are typically measured in saliva only (α-amylase, cortisol, and total salivary IgA) and the remaining four biomarkers (CRP, DHEA, IL-1β, and IL-6) are more commonly measured in serum, but salivary analogs of these serum biomarkers have also been reported. These seven biomarkers were selected based on the availability of immunological or enzymatic assay kits adapted for analysis of saliva as well as their previous applications in environmental health research at EPA [21–24]. Total salivary IgA was measured using a previously developed in-house Luminex assay [25], while the remaining six biomarkers were measured using assay kits from Salimetrics Corp. (Irvine, CA).

Cortisol is a hormone released in response to hypothalamic-pituitary axis activation. Salivary cortisol is extensively used in stress research [26, 27], and it is a common biomarker of AL [13]. It exhibits a diurnal pattern with a pronounced post awakening peak [28]. The amplitude of this peak (cortisol awakening response or CAR), the rate of subsequent decline, and the total cortisol area under the diurnal curve have been used as biomarkers of the stress response [29]. A pronounced morning peak followed by a steep decline with a low level at the end of the day is a healthy pattern, while a relatively flat pattern with a high average level is indicative of chronic stress.

Salivary α-amylase is an enzyme involved in the digestion of carbohydrates. This biomarker of the autonomic nervous system (ANS) reflecting sympathetic arousal and acute stress response [30] was used as a component biomarker of AL in research on the burnout syndrome [31] and in chronic stress research [32]. Its level is usually high at awakening followed by a rapid decline within 30 min and a gradual increase during the rest of the day towards evening maximum. Overall, this biomarker does not vary diurnally as much as salivary cortisol [33, 34].

Salivary IgA is a biomarker of immune function that is under strong neuroendocrine control [35]; it is known to increase in response to transient psychological stress [36].

DHEA is a steroid hormone that has pronounced neuroprotective, antioxidant, and anti-inflammatory effects. Low DHEA levels are indicative of stress and are associated with aging-related conditions, depression, and anxiety-spectrum disorders [37] as well as negative mood [38]. Exposure to dioxins has been linked to reduced salivary DHEA levels [39].

CRP is an acute phase protein that is secreted by the liver in response to infection, trauma, or other inflammatory stimuli [40]. Salivary CRP has been validated against serum CRP [41], and it is an informative biomarker of systemic inflammation [42]. A typical diurnal pattern of salivary CRP involves a peak at awakening [43].

The proinflammatory cytokines interleukin (IL)-1β and IL-6 filter into saliva from serum and are also produced in the oral cavity [40]. Their concentrations in saliva are greater than in serum; salivary and serum concentrations are moderately correlated [44]. Salivary IL-1β and IL-6 increase in response to acute stress [9]. Salivary levels of IL-1β exhibit a pronounced awakening peak with a flat pattern during the day [45]. A diurnal pattern of salivary IL-6 is less pronounced; it may include a minor morning peak, a trough in mid-day, and an increase in the evening [43, 45]. Serum IL-1β and IL-6 have been included in biomarker-based indices of AL [21, 22]. To our knowledge, no previous studies used salivary IL-1β and IL-6 as component biomarkers of AL.

## Methods

### Recruitment and data collection

This pilot cross-sectional survey involved adult (at least 18 years of age and capable of communicating in English) residents of two subsidized housing projects in Denver, Colorado, which were developed in the 1950s and lacked access to amenities and opportunities for indoor/outdoor recreation. The main goal of this pilot survey was to demonstrate recruitment and data collection approaches for characterizing sub-clinical cumulative health impacts of social and behavioral factors in this understudied and difficult to reach population.

After initial consultations with the Denver Housing Authority (DHA) and community representatives, it was determined that noninvasive saliva samples would be the only acceptable form of sample collection for biomarker measurement. Therefore, the aims of the pilot survey were to demonstrate the feasibility of unsupervised saliva sampling in this population, measure IPDI biomarkers, and evaluate their associations with social stressors and self-reported health conditions. The survey was conducted from January 4th to October 29th, 2021 during the COVID-19 quarantine. Because of the quarantine, the original approach involving door-to-door recruitment visits was replaced with recruitment through community advertisements. This modified approach substantially reduced enrollment. While the total target number of participants for this pilot survey was 100 based on the availability of funds and logistical limitations, only 27 individuals had been enrolled.

The protocol was approved by the EPA Human Subjects Research Review Official and the Institutional Review Board of the Colorado State University, which partnered with the EPA on research in these communities. Participants received a consent form, survey questionnaire, saliva sampling instruction and sampling diary, and five SalivaBio passive drool saliva sampling devices (Salimetrics, 5016.04 & 5004.01) designed to collect unstimulated saliva primarily from the parotid gland, as well as a $50 prepaid debit card as compensation for their time.

To characterize the diurnal pattern of salivary cortisol, samples were collected over 1 day using the following schedule: the 1st sample in 5 min after awakening, the 2nd sample in 30 min, the 3rd sample in 2 h, the 4th sample in 5 h, and the 5th sample in 10 h after awakening. Study participants were instructed to refrain from consuming alcohol until after collecting the last sample, and to refrain from eating or drinking until after collecting the second saliva sample. As actual sampling times could deviate from this schedule, study participants were asked to complete a diary that included their wake-up time and actual sample collection times. They were also asked to record episodes of physical exertion and acute stress within one hour prior to sample collection.

Participants were also asked to complete a brief questionnaire in English covering sociodemographic characteristics, height and weight, smoking status, self-reported recent acute illnesses, self-reported chronic diseases and health conditions including allergies, major stressful events during the previous 12 months, such as the loss of employment, illness, or death in the family, as well as their normal physical activity levels and sleeping problems including snoring, sleep apnea, and insomnia. The questionnaire also contained a short 21-question version of the standard Depression, Anxiety, and Stress Scale (DASS-21) survey [46], including depression (mood, motivation, and self-esteem), anxiety (physiological arousal, perceived panic, and fear), and stress (tension and irritability) categories. Category-specific scores were calculated using the standard DASS methodology [47].

Because salivary biomarkers can be degraded at room temperature [10], study participants were instructed to freeze each saliva sample in a household freezer immediately after sampling. The participants then handed their samples and completed survey forms to a DHA employee at a local community center. The samples were stored in a freezer and then shipped to a US EPA laboratory overnight on dry ice in batches. Upon delivery to the laboratory, the samples were aliquoted and stored at −80 °C until analysis.

### Laboratory analyses

ELISA kits from Salimetrics Corp. were used for analysis of salivary cortisol (catalog # 1-3002), CRP (1-2102), DHEA (1-1202), IL-1β (1-3902), and IL-6 (1-3602); a kinetic assay from the same company was used for analysis of salivary α-amylase (1-1902). All tests were conducted in accordance with the manufacturer’s instructions. Samples were diluted in assay buffers prior to analysis. Lower and upper limits of quantitation for each assay are presented in Table 1. Per manufacturer’s instructions, samples with α-amylase and CRP results below the manufacturer-defined lower limits of quantitation (LLOQs) or above the upper limits of quantitation (ULOQs) were reanalyzed, when possible, at a lower or higher dilution, respectively. Final results which remained below LLOQ or above ULOQ were replaced with the LLOQ divided by the square root of two or the ULOQ multiplied by the square root of two, respectively – a common single-imputation method [48]. These imputations did not have any effects on percentile ranks of biomarker values, dichotomization cutoffs, IPDI values, and regression analysis results.

**Table 1:** Summary of immune and neuroendocrine biomarker data

A. Biomarker concentrations in individual samples															
Biomarker	Number of samples	Min	25th pctl	50th pctl	Geom. mean	Mean	75th pctl	Max	IQR	SD	Lower LOQ	N below lower LOQ	Upper LOQ	N above upper LOQ	Mean within-person SD
α-amylase (U/mL)	97	0.05	33	60	47	76	96	566	63	75	0.07*	1	400	1	42
Cortisol—all samples (µg/dL)	97	0.01	0.12	0.20	0.18	0.24	0.30	0.95	0.18	0.17	0.01	0	4.2	0	0.11
Cortisol background (samples 3–5) (µg/dL)	57	0.01	0.10	0.16	0.15	0.19	0.23	0.59	0.13	0.13	0.01	0	4.2	0	0.06
CRP (pg/mL)	94	34	319	648	687	1,227	2,140	4,525	1,821	1,256	18	0	1,600 or 3,200**	21	566
DHEA (pg/mL)	90	12	76	157	142	209	273	989	196	197	7.2	0	1,414	0	81
IgA (mg/dL)	95	0.04	2.8	4.7	4.3	5.8	7.3	21	4.6	4.3	0.0003	0	566	0	2.8
IL-1β (pg/mL)	88	33	61	238	242	592	876	4,243	814	803	47	17	3,000	2	392
IL-6 (pg/mL)	88	5.5	5.5	5.5	9.2	17	9.2	172	3.7	28	7.8	64	500	0	9.7

B. Mean person-level biomarker values										
Biomarker	Number of observations	Min	25th pctl	50th pctl	Geom. mean	Mean	75th pctl	Max	IQR	SD
α-amylase (U/mL)	20	4.7	47	69	57	76	98***	178	51	48
Cortisol background (µg/dL)	20	0.02	0.12	0.18	0.15	0.19	0.22***	0.56	0.11	0.12
CRP (pg/mL)	20	40	535	958	803	1,251	1,726***	4,525	1,190	1,100
DHEA (pg/mL)	20	24	69***	178	143	199	236	622	167	159
IgA (mg/dL)	20	0.10	0.77	1.4	1.4	2.2	3.4***	6.4	2.6	1.8
IL-1β (pg/mL)	20	33	117	449	307	613	907***	1,939	790	629
IL-6 (pg/mL)	20	5.5	5.5	5.5	10	17	14***	80	9.0	23

^*^This is the LLOQ at 1:10 dilution. Samples were initially tested at a 1:200 dilution (LLOQ = 1.4 U/ml); then all samples with α-amylase concentration below the initial LLOQ were retested at a 1:10 dilution (LLOQ = 0.07 U/mL) as per manufacturer’s instruction. One sample was still below the LLOQ after retest (reported concentration 0.05 U/mL)

^**^Samples were initially tested at 1:2 dilution (ULOQ = 1,600 µg/dL). Samples with CRP above the ULOQ were retested at a 1:4 dilution (ULOQ = 3,200 µg/dL) per manufacturer’s instruction, except for 8 samples which were assayed on the last plate; 13 samples were still above the ULOQ after retest (reported concentration 4,525 µg/dL)

^***^Dichotomization cutoff

An in-house total salivary IgA test was conducted using an-house Luminex assay as described previously [25]. Luminex MagPlex® microspheres were coupled to primary goat-anti-human IgA(α) antibody (Seracare, 5210-0154) using the standard Luminex carbodiimide protein coupling protocol (https://info.luminexcorp.com/en-us/research/download-the-xmap-cookbook). A biotinylated version of the same antibody (Seracare, 5260–0027) was used as a secondary antibody along with a streptavidin–phycoerythrin (SAPE) fluorescent label (Invitrogen, S866). Each microplate included serial dilutions of a purified human IgA standard (Jackson ImmunoResearch, 009-000-011). Samples were diluted 1:40,000 in the phosphate buffered saline with 1% bovine serum albumin (Signa Aldrich, A2934) assay buffer and analyzed on an LX-200 plate reader.

For all biomarker tests, at least 20% of samples were assayed as duplicates on the same plate or on different plates. The maximum acceptable intra-plate and inter-plate coefficients of variation were 10% and 25%, respectively.

### Data processing and statistical analysis

Data analysis was conducted using SAS version 9.4 (SAS Institutes). To determine the concentrations of biomarkers (except α-amylase), weighted four-parameter logistic curves were fitted to data on serially diluted standards. For α-amylase, concentrations were calculated using a manufacturer-provided formula. Body Mass Index (BMI) values were calculated from height and weight values reported in questionnaires.

The concentrations of biomarkers in individual samples and numbers of samples tested for 20 individuals included in the final analysis are summarized in Table 1. For all biomarkers except cortisol, the mean concentration from all available samples was used in further analysis (Table 1). For cortisol, two approaches were considered for summarizing the data: (i) the amplitude of the post-awakening peak and (ii) the background level calculated as the mean of samples 3, 4, and 5 (excluding the morning peak). Cortisol time series plots (Supplementary Fig. S1) demonstrated that only six individuals had clearly defined morning peaks; the remaining individuals had rather flat diurnal patterns. This could be due to study participants incorrectly reporting their wake-up time or failing to collect samples at exact specified post awakening intervals, which was especially important for the first two morning samples. At the same time, the background cortisol levels exhibited substantial inter-person variability. Therefore, the background cortisol level (mean of samples 3 - 5) was used in further analysis and as a component biomarker of IDPI.

Data on all biomarkers were then dichotomized at distribution-based cutoff points (Table 1), with a value of “1” assigned to the values above the 75th percentile for α-amylase, background cortisol, CRP, IL-1β, IL-6, and total IgA (high levels are known to be associated with chronic stress) and below the 25th percentile for DHEA (low levels associated with chronic stress).

Associations between questionnaire variables and individual dichotomized biomarkers were analyzed using logistic regression models adjusted for age. Age was selected due to the pre-existing knowledge about its effects on these biomarkers [21, 22] as well as preliminary analysis which showed that age was a consistent predictor of biomarker values. Due to the small sample size, no other covariates could be included in the regression models. The results are presented as age-adjusted odds ratios of having a potentially unhealthy biomarker value.

IDPI was estimated as the sum of dichotomized biomarkers and represented the total number of biomarkers with potentially unhealthy values—a Poisson-distributed count variable. Analysis of associations between self-reported stress factors and IPDI was conducted using Poisson regression models adjusted for age. The results are presented as multiplicative changes in the mean IPDI.

Due to the small sample size and the exploratory nature of this pilot study, all regression results with *p* < 0.2 were reported as indicative of potential associations. The *p* = 0.2 cut-off was selected because it is commonly used for selecting covariates for multi-variate logistic regression models [49, 50].

## Results

Out of 27 individuals enrolled in the study, 20 (74%) individuals properly completed data collection procedures and provided valid saliva samples of sufficient volume for biomarker tests. Their average age was 46 years (range from 18 to 74 years); 30% were taking medicine for depression, 45% were obese (BMI ≥ 30 kg/m^2^), 40% reported insomnia, and 65% reported having various chronic health conditions, including diabetes (30%), allergies to house dust (35%), and allergies to pollen (50%) (Table 2). Five individuals (25%) reported losing their employment during the previous 12 months.

**Table 2:** Descriptive statistics of the study population (N = 20)

Variable	Level	Number of individuals	Percent
Race	Not reported	1	5
	American Indian	2	10
	Black	3	15
	Pacific Islander	1	5
	Mixed Race	6	30
	White	7	35
Ethnicity	Hispanic	8	40
	Non-Hispanic	10	50
	Not reported	2	10
Sex	Male	4	20
	Female	15	75
	Not reported	1	5
Allergy	No allergy	8	40
	Any allergy	12	60
	Allergy to dust	7	35
	Allergy to medicine	4	20
	Allergy to pollen	10	50
Obesity (BMI ≥ 30 kg/m^2^)	No	9	45
	Yes	9	45
	Missing data	2	10
Chronic illness	No illness	7	35
	Any chronic illness	13	65
	Diabetes	6	30
	High blood pressure	7	35
Anxiety medicine	No	15	75
	Yes	4	20
	Not reported	1	5
Depression medicine	No	13	65
	Yes	6	30
	Not reported	1	5
Insomnia	No	11	55
	Yes	8	40
	Not reported	1	5
Current smoker	No	14	70
	Yes	6	30
Past smoker (excluding current smokers)	No	9	45
	Yes	5	25

Most participants (90%) reported that they were comfortable with saliva sampling, although collecting five samples during a day according to the specified schedule appeared to be a challenging task. Fewer participants (75%) reported that they remembered collecting all required saliva samples on time.

The concentrations of salivary biomarkers (Table 1) were generally consistent with previously published data [11, 44]. All individual measurements of cortisol, DHEA, and total IgA were within the corresponding assay quantitation ranges. Over half of the IL-6 measurements (64 of 88 samples, 73%) were below the LLOQ. For all biomarkers, the mean within-person standard deviation was smaller than the overall standard deviation for all samples analyzed (Table 1). IPDI values varied from 0 to 5 (mean 1.75, median 1) and were approximately Poisson-distributed.

Associations between questionnaire variables and dichotomized biomarkers with *p* < 0.2 (29 associations) are presented in Table 3. Of these, 27 associations suggested increased odds of having potentially unhealthy biomarker values in individuals who were exposed to stress factors or had chronic health conditions. The remaining two associations were between the presence of children in the household and increased odds of high salivary α-amylase and between alcohol consumption and increased odds of having a low DHEA level. Stress factors and health conditions associated with unhealthy levels of various biomarkers included the loss of employment, self-reported difficulties adjusting to limitations due to the COVID-19 pandemic, increased DASS-21 stress score, obesity, diabetes, depression, insomnia, and allergies to dust, pollen, or medicines. Allergies tended to be associated with high levels of the inflammation biomarkers (CRP, IgA, IL-1β, and IL-6), while the loss of employment was associated with unhealthy levels of α-amylase and cortisol, which are markers of sympathetic nervous system activation.

**Table 3:** Strongest (*p* < 0.2) associations between questionnaire variables and salivary biomarkers of the neuroendocrine and immune systems: odds ratios of unhealthy biomarker values with 95% confidence limits

Outcome	Predictor	Number of observations	Odds ratio of unhealthy biomarker level*	P value
α-amylase	Children are present in the household	19	2.79 (0.99; 7.86)	0.05
α-amylase	Loss of employment during past year	19	4.13 (0.68; 25.0)	0.12
α-amylase	Diabetes	20	3.91 (0.53; 28.8)	0.18
α-amylase	Difficulty adjusting to COVID-19 rules	20	3.56 (0.55; 23.2)	0.18
Cortisol level	Takes medicine for depression	19	6.43 (0.67; 61.9)	0.11
Cortisol level	Loss of employment during past year	19	4.41 (0.70; 27.6)	0.11
Cortisol level	Treated for depression	19	6.22 (0.64; 60.3)	0.11
Cortisol level	Age, per 1 year increase	20	1.06 (0.99; 1.13)	0.12
Cortisol level	Takes medicine for anxiety	19	3.66 (0.52; 26.0)	0.19
CRP	Allergy to house dust requiring treatment	20	5.72 (0.95; 34.33)	0.06
CRP	Allergy to house dust	20	8.00 (0.77; 82.90)	0.08
CRP	Allergy to pollen requiring treatment	20	4.18 (0.69; 25.38)	0.12
CRP	Self-reported chronic obesity	20	3.68 (0.61; 22.02)	0.15
CRP	Visited doctor due to a health issue	20	3.44 (0.55; 21.33)	0.18
DHEA	Age, per 1 year increase	20	1.09 (1.00; 1.18)	0.04
DHEA	Recreational alcohol use	18	4.70 (0.63; 35.0)	0.13
IgA	Allergy to house dust	20	12.7 (1.23; 132)	0.03
IgA	Allergy to dust requiring treatment	20	6.53 (1.05; 40.4)	0.04
IgA	Allergy to pollen requiring treatment	20	5.30 (0.81; 34.6)	0.08
IgA	Allergy to pollen	20	5.39 (0.55; 52.7)	0.15
IgA	Self-reported chronic obesity	20	3.45 (0.57; 20.9)	0.18
IL-1β	Allergy to house dust	20	11.4 (1.10; 118)	0.04
IL-1β	Allergy to medicine	20	6.05 (1.00; 36.7)	0.05
IL-1β	Diabetes	20	4.40 (0.57; 34.1)	0.16
IL-1β	Allergy to pollen	20	4.98 (0.51; 49.1)	0.17
IL-6	Insomnia	19	5.06 (0.64; 40.1)	0.13
IL-6	DASS-21 stress score, per unit increase	17	1.24 (0.94; 1.64)	0.14
IL-6	Sleeping difficulties (any reason)	19	4.72 (0.55; 40.6)	0.16
IL-6	Allergy to dust requiring treatment	20	3.50 (0.54; 22.6)	0.19

^*^Effect estimates for age are from univariate models; all other effect estimates are adjusted for age

Associations between predictor variables and IPDI with *p* < 0.2 are presented in Table 4. Allergy to house dust was significantly (*p* < 0.05) associated with 2.11 (1.00; 4.41)-fold greater mean IPDI, adjusted for age. Another marginally significant association was with allergy to house dust requiring treatment. The remaining ten associations included greater age (from a univariate model), allergies to pollen, obesity (both self-reported and determined using BMI), diabetes, loss of employment, and greater DASS-21 scores for stress, anxiety, and depression. All 12 associations suggested increased IPDI in individuals who reported exposure to stressors or health conditions.

**Table 4:** Strongest (*p* < 0.2) associations between predictor variables and the saliva-based Inflammation and Physiological Dysregulation Index (IPDI), multiplicative effects with 95% confidence limits

Predictor variable	Number of observations	Fold change in IPDI*	P value
Allergy to house dust	20	2.11 (1.00; 4.41)	0.05
Allergy to house dust requiring treatment	20	2.07 (1.00; 4.28)	0.05
Age (per 10-year increase)	20	1.23 (0.99; 1.54)	0.06
Allergy to pollen requiring treatment	20	1.91 (0.92; 3.95)	0.08
DASS-21 stress score, per unit increase	17	1.11 (0.99; 1.25)	0.08
Self-reported chronic obesity	20	1.81 (0.92; 3.56)	0.08
Diabetes	20	1.67 (0.83; 3.34)	0.15
DASS-21 anxiety score, per unit increase	16	1.09 (0.97; 1.22)	0.15
Loss of employment during past year	19	1.65 (0.83; 3.29)	0.16
BMI, kg/m^2^ (per unit increase)	18	1.04 (0.98; 1.10)	0.16
Obesity (BMI ≥ 30 kg/m^2^)	18	1.70 (0.81; 3.56)	0.16
DASS-21 depression score, per unit increase	16	1.06 (0.98; 1.14)	0.17

^*^Effect estimate for age is from a univariate model; all other effect estimates are adjusted for age

## Discussion

This pilot study demonstrated the use of self-collected noninvasive saliva samples for the analysis of seven biomarkers of the neuroendocrine and immune functions. Results of regression analysis suggested that unhealthy levels of individual biomarkers and increased IPDI were associated with self-reported exposure to chronic social stressors and to health conditions which are known to adversely affect the neuroimmune system. To the best of our knowledge, the use of a salivary IPDI is a novel noninvasive approach. Allergy to house dust was significantly associated with increased IPDI and potentially unhealthy levels of total IgA and IL-1β. Multiple associations with IPDI and its component biomarkers that were short of being statistically significant (0.05 < *p* < 0.2) were all suggestive of the detrimental effects of age, allergies, obesity, depression, insomnia, loss of employment, and difficulties adjusting to COVID-19 quarantine, as well as DASS-21 depression, anxiety, and stress scores. These effects are consistent with the existing knowledge on risk factors of physiological dysregulation and systemic inflammation.

Specifically, house dust mites are the most common cause of allergic symptoms and a risk factor for asthma [51]. Immune responses to dust mite antigens involve allergic inflammation, the secretion of proinflammatory cytokines [52], and increased levels of IL-1β [53] and IL-6 [54]. Exposure to pollen is also associated with allergic rhinitis and inflammation in susceptible individuals [55, 56]. Age, obesity, and high BMI are associated with increased AL and chronic inflammation [57–61]. Sleep disturbances have also been linked to AL [62]. Chronic stress and stressful life events are known factors that impact biomarkers of AL and inflammation [13, 18, 63–65]. Finally, psychological stress from COVID-19 quarantine has been linked to potentially unhealthy levels of salivary cortisol and α-amylase [66]. This study did not produce evidence of associations of sex and smoking with IPDI and component biomarkers, which is consistent with previous studies using similar biomarkers and composite indices of AL and inflammation [21, 22].

Although many of associations reported above were not statistically significant, the fact that all of them were in the direction consistent with existing knowledge provides evidence of the usability of individual salivary biomarkers and IPDI as noninvasive measures of the cumulative sub-clinical health impacts of diverse social, behavioral, and environmental stressors. This approach should be further validated in a larger study to confirm these preliminary findings.

## Limitations

The main limitation of this pilot study is its small sample size (20 individuals) resulting in the low statistical power. Regression models could only be adjusted for age, as there should be at least 10 observations per covariate [67]. Therefore, some of the observed associations could be affected by unaccounted confounding factors. Another limitation is the limited set of neuroendocrine and immune biomarkers. The study which did not include some core biomarkers of AL which could not be measured in saliva. Some salivary biomarkers known to respond to stress, such as aldosterone, were not included because of the limited scale of this pilot study. Awakening and sampling times were self-reported by study participants which could result in imprecise data. Finally, immunoassay-based salivary biomarkers were not compared to gold standard measurements but evaluated indirectly through demonstrating their associations with known risk factors for chronic inflammation and physiological dysregulation.

## Supplementary Information

Supplementary material 1.

## Data Availability

Study data cannot be shared publicly because of Personally Identifiable Information (PII) on study participants. EPA cannot release PII regarding living individuals according to the Privacy Act and the Freedom of Information Act (FOIA). Further information about requesting access to these data for researchers who meet the criteria for access to confidential data will be posted after the publication of this manuscript at https://catalog.data.gov/organization/epa-gov.
