# Supplementary figures and images for: Pilot application of an inflammation and physiological dysregulation index based on noninvasive salivary biomarkers
Source: BMC Res Notes. 2025 Feb 5;18:53. doi: 10.1186/s13104-024-07056-4 (PMC11796071; doi:10.1186/s13104-024-07056-4)

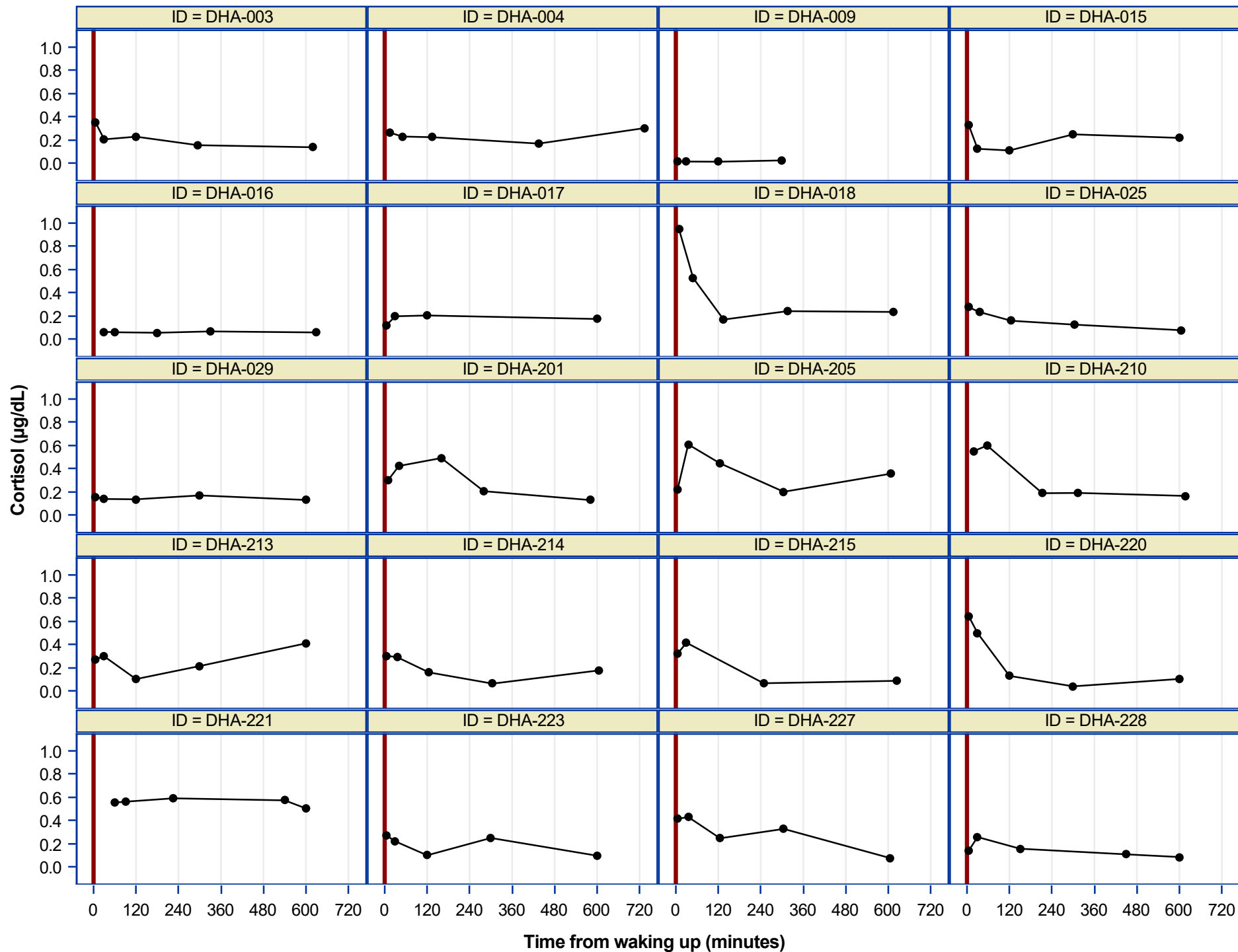

Supplement: Supplementary file 1 — Supplementary material 1. [file 13104_2024_7056_MOESM1_ESM.pdf]
